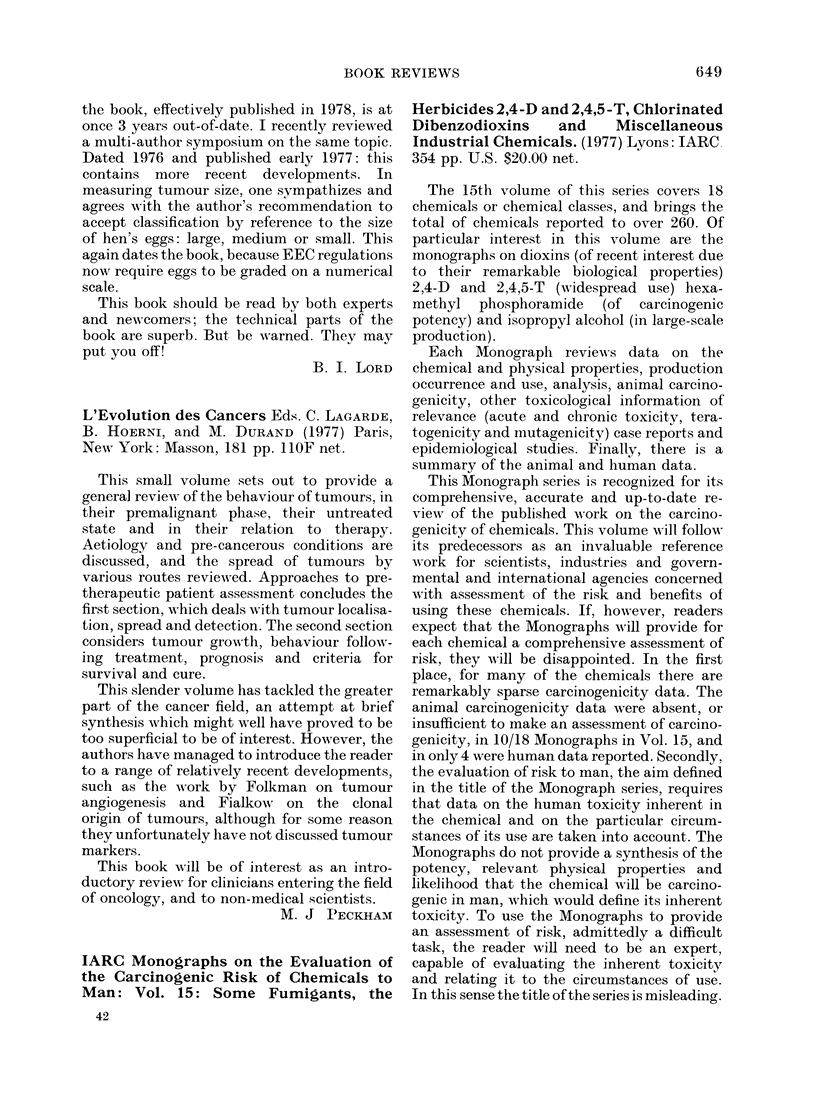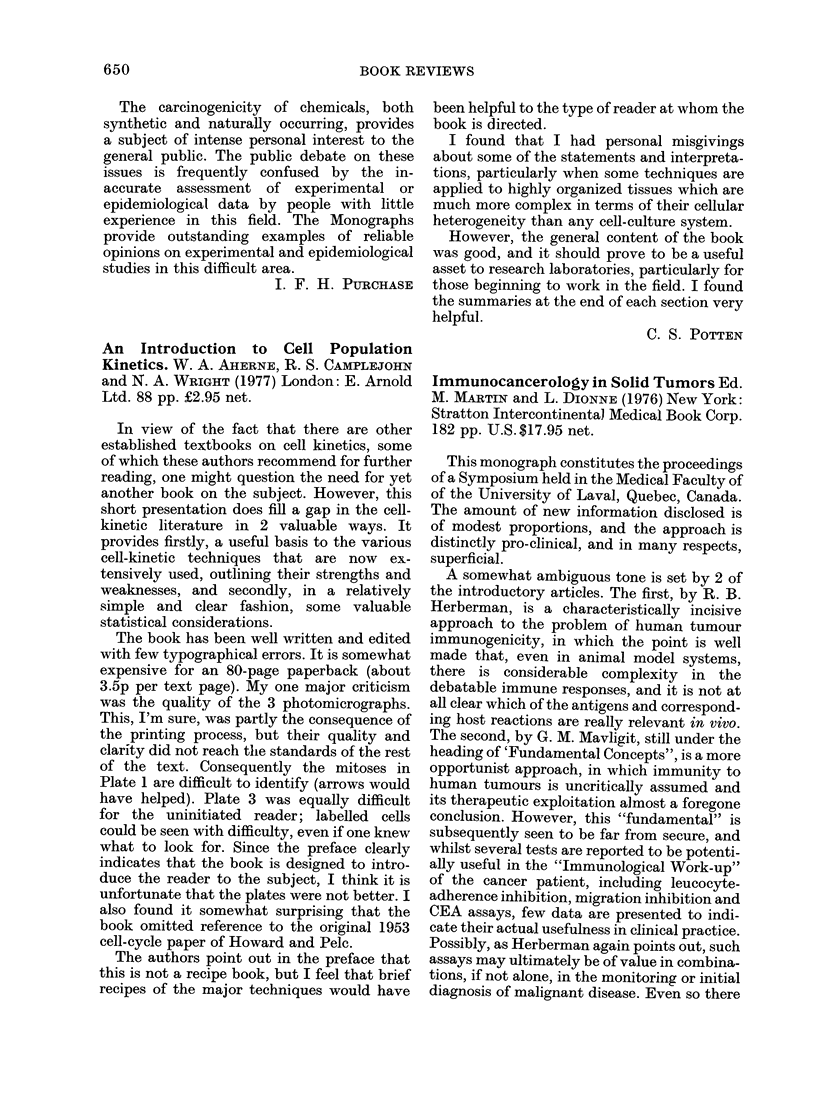# IARC Monographs on the Evaluation of the Carcinogenic Risk of Chemicals to Man: Vol. 15: Some Fumigants, the Herbicides 2,4-D and 2,4,5-T, Chlorinated Dibenzodioxins and Miscellaneous Industrial Chemicals

**Published:** 1978-04

**Authors:** I. F. H. Purchase


**IARC Monographs on the Evaluation of the Carcinogenic Risk of Chemicals to Man: Vol. 15: Some Fumigants, the Herbicides 2,4-D and 2,4,5-T, Chlorinated Dibenzodioxins and Miscellaneous Industrial Chemicals.** (1977) Lyons: IARC. 354 pp. U.S. $20.00 net.

The 15th volume of this series covers 18 chemicals or chemical classes, and brings the total of chemicals reported to over 260. Of particular interest in this volume are the monographs on dioxins (of recent interest due to their remarkable biological properties) 2,4-D and 2,4,5-T (widespread use) hexamethyl phosphoramide (of carcinogenic potency) and isopropyl alcohol (in large-scale production).

Each Monograph reviews data on the chemical and physical properties, production occurrence and use, analysis, animal carcinogenicity, other toxicological information of relevance (acute and chronic toxicity, teratogenicity and mutagenicity) case reports and epidemiological studies. Finally, there is a summary of the animal and human data.

This Monograph series is recognized for its comprehensive, accurate and up-to-date review of the published work on the carcinogenicity of chemicals. This volume will follow its predecessors as an invaluable reference work for scientists, industries and governmental and international agencies concerned with assessment of the risk and benefits of using these chemicals. If, however, readers expect that the Monographs will provide for each chemical a comprehensive assessment of risk, they will be disappointed. In the first place, for many of the chemicals there are remarkably sparse carcinogenicity data. The animal carcinogenicity data were absent, or insufficient to make an assessment of carcinogenicity, in 10/18 Monographs in Vol. 15, and in only 4 were human data reported. Secondly, the evaluation of risk to man, the aim defined in the title of the Monograph series, requires that data on the human toxicity inherent in the chemical and on the particular circumstances of its use are taken into account. The Monographs do not provide a synthesis of the potency, relevant physical properties and likelihood that the chemical will be carcinogenic in man, which would define its inherent toxicity. To use the Monographs to provide an assessment of risk, admittedly a difficult task, the reader will need to be an expert, capable of evaluating the inherent toxicity and relating it to the circumstances of use. In this sense the title of the series is misleading.

The carcinogenicity of chemicals, both synthetic and naturally occurring, provides a subject of intense personal interest to the general public. The public debate on these issues is frequently confused by the inaccurate assessment of experimental or epidemiological data by people with little experience in this field. The Monographs provide outstanding examples of reliable opinions on experimental and epidemiological studies in this difficult area.

I. F. H. PURCHASE